# Biomechanical and Physiological Demands of CrossFit: A Systematic Review

**DOI:** 10.5114/jhk/214743

**Published:** 2026-03-30

**Authors:** Alexandra Malheiro, Pedro Forte, David Rodríguez-Rosell, Diogo Luís Marques, Mário Cardoso Marques

**Affiliations:** 1 Department of Sport Sciences, University of Beira Interior, Covilhã, Portugal.; 2 Research Center in Sports Sciences, Health Sciences, and Human Development (CIDESD), Covilhã, Portugal.; 3 Department of Sports, Instituto Politécnico de Bragança, Bragança, Portugal.; 4 Department of Sports, Higher Institute of Educational Sciences of the Douro, Penafiel, Portugal.; 5 Research Center for Active Living and Wellbeing (LiveWell), Instituto Politécnico de Bragança, Bragança, Portugal.; 6 Physical Performance and Sports Research Center, Universidad Pablo de Olavide, Seville, Spain.; 7 Department of Sport and Informatics, Universidad Pablo de Olavide, Seville, Spain.

**Keywords:** fitness, physical performance, biomechanics, physiology, high-intensity interval training

## Abstract

CrossFit’s popularity has increased as an effective training program for physical fitness. The volume of published literature suggests a continuous effort to understand and optimize CrossFit training protocols. Therefore, this study aimed to analyze scientific literature findings related to CrossFit’s biomechanical and physiological demands via a systematic review. Systematic searches were conducted on PubMed, Web of Science, ScienceDirect, Scopus, and SciELO databases for articles reporting the effects of CrossFit training. Following the PRISMA guidelines, nineteen studies (n = 537 participants) examined the use of biomechanical and/or physiological variables in CrossFit performance. This review considered the one-repetition maximum, the countermovement jump, peak power, and movement technique as biomechanical variables most often used in literature. The physiological variables included blood lactate, maximal oxygen uptake, heart rate variability, and the rating of perceived exertion. These variables accurately measured strength, aerobic and anaerobic capacity, along with fatigue in training sessions and competitions. CrossFit training was shown to improve maximal oxygen uptake, muscle strength, hypertrophy, and muscular endurance while also inducing physiological stress. Strength and power variables correlated strongly with CrossFit performance, but movement technique and postural control also played significant roles. The combination of aerobic and anaerobic elements within CrossFit enhanced cardiovascular fitness and anaerobic capacity, reinforcing effectiveness when appropriately managed.

## Introduction

The increasing popularity of CrossFit as a sport has likely contributed to a significant increase in scientific research (Dominski et al., 2021; Meier et al., 2023; Rodríguez et al., 2022). The exponential growth of CrossFit is unprecedented and signified by over 15,000 CrossFit-affiliated gyms worldwide and the global recognition as a “fitness sport” (Dexheimer et al., 2019; Dominski et al., 2021; Sauvé et al., 2024), with over 5 million athletes worldwide engaging in CrossFit-based training (Gianzina and Kassotaki, 2019; Meier et al., 2023). A review of the available literature reveals a substantial number of publications addressing various aspects of CrossFit (Claudino et al., 2018; Jacob et al., 2020; Mehrab et al., 2023; Meier et al., 2023), including exercise programming (Ambroży et al., 2022; Knapik, 2015; Oliver-López et al., 2024), injury prevention (Klimek et al., 2018; Nicolay et al., 2022; Shim et al., 2023), performance optimization (Shaw and Sergent, 2019; Sousa et al., 2016; Tafuri et al., 2019), and metabolic demands during different workout configurations (Feito et al., 2019; Gómez-Landero and Frías-Menacho, 2020; Maté-Muñoz et al., 2017).

CrossFit methodology is widely classified under high-intensity functional training, which is defined by the use of functional, multi-joint movements performed at high intensity and incorporating both aerobic and resistance exercise modalities (Feito et al., 2019; Heinrich et al., 2015). Indeed, CrossFit is recognized as a high-intensity sport involving powerlifting and gymnastics movements using heavy loads and maximal efforts (Dominski et al., 2021; Fisker et al., 2017; Tibana et al., 2018). Therefore, improvements in physical fitness are based on strength and conditioning training using complex, powerful, and continuous movements that stimulate practitioners’ metabolism exponentially (Maté-Muñoz et al., 2017; Schlegel, 2020; Tibana et al., 2021). Due to the high intensity of the exercises used and short or no rest periods, reduced movement velocity during exercise (i.e., fatigue) is considered a determining factor (Faelli et al., 2020; Maté-Muñoz et al., 2017). In the literature, fatigue has been defined as an involuntary loss of muscle strength across successive repetitions, leading to a notable reduction in movement velocity during exercise execution (Banyard et al., 2017; Maté-Muñoz et al., 2017; Sánchez-Medina and González-Badillo, 2011). Given that CrossFit is characterized by highly varied workouts often performed near or to the point of muscular failure, understanding patterns of velocity loss, commonly used as a proxy for neuromuscular fatigue (González-Badillo et al., 2022; Sánchez-Medina and González-Badillo, 2011), may serve as a valuable indicator for performance optimization, injury prevention, and training load monitoring.

Generally, training sessions are organized as daily sessions named workout of the day (WOD). These WODs are typically scheduled to perform the exercises as fast as possible, i.e., “for time” (FT), or to perform the maximum number of repetitions or rounds in an established time, i.e., “as many rounds as possible” (AMRAP) (Glassman, 2010; Menargues-Ramírez et al., 2022). The athletes’ progression in CrossFit could be measured through these sessions, provided that the execution conditions are controlled (including exercises, loads, repetitions, or execution time). However, due to the constant variations introduced in each WOD, it is important to track changes through periodic monitoring of the performance of specific exercises. Therefore, benchmark WODs are established to assess the progress of some workouts by comparing the number of repetitions and time for completion over time and between athletes (Glassman, 2003). For example, the benchmark WOD “Fran” consists of performing 21-15-9 repetitions for time of pull-ups and thrusters, and “Grace” is a set of 30 clean and jerks to complete for time (Glassman, 2003). In this regard, previous studies have investigated the influence of different factors on the performance in benchmark WODs, in ‘CrossFit Open’ workouts, or the placement in the ‘CrossFit Games’ (Carreker and Grosicki, 2020; Fernández-Fernández et al., 2015; Kliszczewicz et al., 2014; Maté-Muñoz et al., 2017). Evidence-based recommendations for effective and specific training programming could be developed by identifying performance predictors, leading to optimal competitive performance.

Several studies have investigated biomechanical aspects of CrossFit exercises, focusing on movement patterns, joint loading, and muscular activation (Cejudo, 2022; Ferreira et al., 2020; Martínez-Gómez et al., 2019). At the core of these considerations there are joint kinematics which describe movement patterns of joints during various exercises, notably Olympic lifts. Understanding these patterns is crucial, as they have been demonstrated to impact both performance outcomes and injury risk among CrossFit practitioners (Butcher et al., 2015b; Naderi et al., 2025). For instance, Butcher et al. (2015b) observed that biomechanical efficiency tended to decline under fatigue during high-repetition strength exercises, with a corresponding decrease in performance.

Beyond joint movement, neuromuscular activation also plays a vital role in biomechanical efficiency during CrossFit. Adequate levels of muscular strength are essential for executing complex, multi-joint movements such as Olympic lifts. In this context, Martínez-Gómez et al. (2019) demonstrated strong correlations between lower-body power, as measured by full squat performance, and success in CrossFit workouts. Their findings suggest that biomechanical proficiency not only supports technical execution, but also serves as a key determinant of performance outcomes. This evidence reinforces the relevance of biomechanical assessment for guiding both the training program design and performance monitoring.

Additionally, biomechanical demands in CrossFit extend to aspects such as load management and consistency of the bar path during lifting tasks (Polydorou et al., 2024). Equipment choices can also play a role in optimizing movement (Meyer et al., 2017). For example, Waryasz et al. (2016) found that wearing weightlifting shoes with elevated heels could modify joint angles during lifts, thereby reducing shear stress on the spine. These biomechanical insights inform evidence-based decisions regarding gear selection and technique adjustments, ultimately aiming to improve performance while mitigating injury risk.

Additionally, studies exploring physiological demands of CrossFit training have examined factors such as cardiovascular responses, energy expenditure, and muscle fatigue (Faelli et al., 2020; Mangine et al., 2020). The published literature suggests a continuous effort to understand and optimize CrossFit training protocols to improve performance by delaying fatigue appearance (Mangine et al., 2023; Weisenthal et al., 2014). According to Meyer et al. (2017), one of the most important factors for improving the ability to resist loss of strength or velocity is the proper manipulation of training load and volume. Considering this, WODs often involve a high volume of repetitions across different exercises, which could promote rapid fatigue, particularly when training volume and intensity are not adequately periodized, which may result in a decline in movement quality and an increased risk of injury (Gardiner et al., 2020; Mehrab et al., 2023).

Despite the extensive research conducted over the last decade on CrossFit, there is still no consensus on the key factors that determine performance or on the mechanical and metabolic responses to different WODs. This knowledge could allow for improved training processes for CrossFitters. Therefore, in an attempt to synthesize existing information and continue to improve the scientific research in CrossFit, this systematic review aimed to assess biomechanical and physiological variables and demands related to CrossFit training.

## Methods

### Study Design

To guide the organization of findings, we applied a thematic analysis that grouped studies into two primary domains: biomechanical and physiological. This categorization was informed by the predominant outcomes and assessment tools used across literature. Studies classified as biomechanical primarily investigated variables such as joint kinematics, movement velocity, force production, power output, and muscle activation, often employing technologies such as motion capture, electromyography (EMG), and force platforms (Butcher et al., 2015b; Cejudo, 2022; Martínez-Gómez et al., 2019; Maté-Muñoz et al., 2018; Yüksel et al., 2018). In contrast, physiological studies focused on aspects related to the internal load and fatigue responses, including maximal oxygen uptake (VO_2max_), blood lactate concentration, heart rate variability (HRV), and the rate of perceived exertion (RPE) (Faelli et al., 2020; Schlie et al., 2023; Tibana et al., 2018; Zeitz et al., 2020). The rationale for these thematic divisions aligns with established distinctions in the literature between mechanical and metabolic demands in training (Bachero-Mena and González-Badillo, 2021; Di Michele et al., 2012; Maté-Muñoz et al., 2018; Rios et al., 2024). However, the specific structure of sub-themes in this review was developed inductively, based on the objectives and reported outcomes of the included studies. For instance, outcomes such as the countermovement jump (CMJ) and one-repetition maximum (1RM) were grouped under strength and power performance within the biomechanical dimension. To synthesize the existing information, we considered the biomechanical and physiological aspects discussed above to identify the performance variables in CrossFit training and competition.

### Search Strategy

This systematic literature search followed the Preferred Reporting Items for Systematic Reviews and Meta-Analyses (PRISMA) guidelines (Page et al., 2021). The review protocol was prospectively registered in the International Platform of Registered Systematic Reviews (INPLASY) under the registration number INPLASY202560108. The protocol is publicly available at https://doi.org/10.37766/inplasy2025.6.0108. Studies were retrieved from PubMed, Web of Science, ScienceDirect, Scopus, and SciELO databases. These databases were selected to ensure comprehensive coverage of both international and regional literature relevant to the physiological and biomechanical aspects of CrossFit. While PubMed, Scopus, and Web of Science databases are widely recognized for indexing peer-reviewed scientific publications in health and sports science, ScienceDirect was included due to its accessibility to full-text articles from journals that frequently publish exercise science research. SciELO was added to capture relevant literature from Latin American sources that might not be indexed in other major databases. However, it was acknowledged that not including databases such as SPORTDiscus, Embase, or Google Scholar, might have introduced database selection bias. This limitation should be considered when interpreting the completeness of the included evidence.

Studies were searched using a Boolean string with specific keywords (Table 1). The literature search was performed between December 2023 and February 2024 by an independent author and checked by a second author (P.F.). These procedures were conducted according to the recommended PRISMA guidelines (Page et al., 2021).

**Table 1:** Search terms and keywords used in the screening procedures of the systematic review.

Search term		Keywords
Population	1	“CrossFit” OR “high functional training” OR “functional fitness”
Intervention	2	“effects” OR “training effects” OR “training strategies”
Comparison/outcomes	3	Physiological set: “training load” OR “external training load” OR “internal training load” OR “physical performance” OR “physiological performance” OR “physical response” OR “physical demands” OR “physiological response” OR “physiological demands” OR “activity profile” OR “workload” OR “loading” OR “athletic performance” OR “sports performance” OR “velocity loss” OR “movement speed loss” OR “peak power” OR “power” OR “propulsive velocity” OR “MPV” OR “heart rate” OR “perceived exertion” OR “lactate” OR “acid lactic” OR “volume of oxygen consume” OR “VO2max” OR “metabolic power” OR “energy cost” OR “high intensity” OR “conditioning” OR “fitness” OR “biomechanics” OR “kinetic” OR “kinematic” OR “physiology”
Boolean syntax	4	(((#4) AND #3) AND #2) AND #1

### Inclusion Criteria

The following inclusion criteria were applied: i) peer-reviewed studies with experimental (randomized and non-randomized trials), observational (cohort, case-control), or cross-sectional designs investigating the effects of CrossFit; ii) studies involving adult CrossFit practitioners aged ≥ 18 years; iii) studies conducted with healthy adults, athletes or individuals with specific health conditions actively engaged in CrossFit training; iv) studies assessing physiological or biomechanical variables following CrossFit training or participation, including both intervention-based and descriptive designs (e.g., studies measuring fatigue responses during benchmark WODs without a structured intervention); v) studies providing at least a partial description of the CrossFit program applied, such as training frequency, load, volume, duration, or types of exercise, even if not all training details were fully specified due to ecological or real-world research constrains; and vi) studies reporting quantitative outcomes related to physical fitness, performance, or physiological and biomechanical responses.

### Data Extraction and Coding

To ensure transparency and methodological rigor, the screening and data extraction procedures were conducted following the PRISMA 2020 guidelines (Page et al., 2021). Two independent reviewers (A.M. and P.F.) screened the titles, abstracts, and full texts of all retrieved studies based on the predefined inclusion criteria. Any disagreements during the selection process were resolved through discussion, and when necessary, a third reviewer (D.L.M.) was consulted to reach consensus. Duplicate records were identified and removed using Microsoft Excel, supplemented by manual verification to ensure accuracy. Data extraction was also carried out independently by two reviewers and focused on the following categories: i) sample characteristics, the study design, and the competitive level; ii) performance-related outcomes including measurement variables, thresholds, and formulas; iii) references and foundational studies on which the included articles based their methodology; iv) methodological details such as study aims, experimental procedures, data collection methods, and statistical analysis. This structured and collaborative approach was intended to minimize bias, improve consistency, and strengthen the credibility of the review in line with established reporting standards (Page et al., 2021).

### Study Quality Assessment

Two reviewers independently assessed the methodological quality of the included studies, with discrepancies resolved by consensus. For randomized controlled trials (RCTs), the Physiotherapy Evidence Database (PEDro) scale was applied. This tool consists of 11 items, with each item scored as either 0 or 1, yielding a maximum score of 10 (the first item is not included in the final score). Studies scoring ≥ 6 were considered of high quality, consistent with established cut-offs (García-Pinillos et al., 2017). For cross-sectional studies, the checklist “Joanna Briggs Institute (JBI) Critical Appraisal Checklist for Analytical Cross-Sectional Studies” was used. This checklist includes eight items evaluating aspects such as sample selection, validity of measurements, and appropriateness of statistical analysis (Munn et al., 2023). Each item was rated as “yes”, “no”, “unclear”, or “not applicable”. Studies meeting six or more criteria were of moderate to high methodological quality. No studies were excluded based on methodological quality. Although formal inter-rater reliability (e.g., Cohen’s kappa) could not be calculated due to identical ratings across studies (McHugh, 2012), complete agreement (100%) was observed between reviewers. Any minor uncertainties were resolved through discussion to ensure consistency and transparency throughout the appraisal process.

## Results

The results section follows a presentation based on study searching results, quality of the included studies, and performance indicators. The performance variables were subdivided into biomechanical and physiological.

### Study Search Results

**Figure 1:**
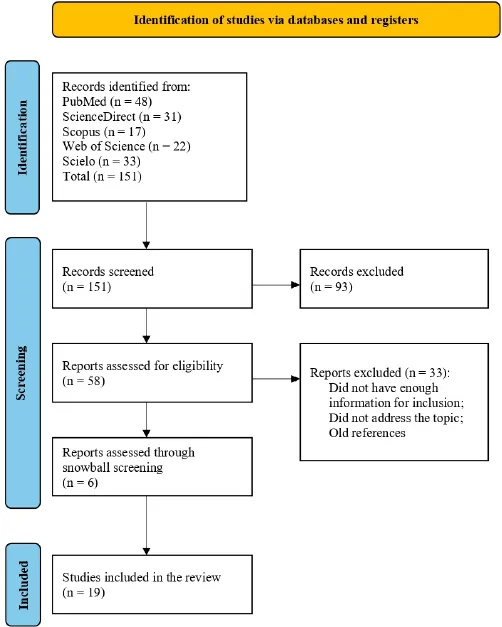
Literature searching flowchart.

The search strategy found 151 articles (48 articles from PubMed, 31 from ScienceDirect, 17 from Scopus, 22 from Web of Science, and 33 from SciELO). Following full-text screening, 19 articles were included. The selection process is outlined in Figure 1, adhering to PRISMA guidelines. To conduct this review, the existing body of literature on CrossFit was first explored to gain a comprehensive understanding of the current research landscape. Based on this synthesis and the defining characteristics of CrossFit, the identified performance-related variables were organized into two overarching categories (physiological and biomechanical dimensions), reflecting how these aspects are commonly addressed in literature. In defining the main themes of this review, priority was given to elements of CrossFit training with strong theoretical and practical relevance for researchers, coaches, and athletes. These include: i) biomechanical variables that characterize movement patterns, joint loading, and mechanical demands during training and competition, and ii) physiological aspects related to muscular fatigue, metabolic stress, and key performance indicators.

### Quality of the Included Studies

In the evaluation of methodological quality, the qualitative scores for 15 cross-sectional studies ranged from 6 (lowest quality) to 7 (highest quality) out of a maximum of 8 possible points in the JBI scale (Table 2). Fourteen studies received a score of 6, while one study had a score of 7. Regarding the experimental studies, four studies scored 9 (highest quality) out of 11 points on the PEDro scale (Table 3).

**Table 2:** Study quality analysis using the Joanna Briggs Institute (JBI) Critical Appraisal Checklist for Analytical Cross-Sectional Studies.

Authors	Q1	Q2	Q2	Q4	Q5	Q6	Q7	Q8	Total
Brognara et al. (2023)	Y	Y	Y	Y	N	N	Y	Y	6/8
Butcher et al. (2015a)	Y	Y	Y	Y	N	N	Y	Y	6/8
Butcher et al. (2015b)	Y	Y	Y	Y	N	N	Y	Y	6/8
Cejudo (2022)	Y	Y	Y	Y	N	N	Y	Y	6/8
Faelli et al. (2020)	Y	Y	Y	Y	N	N	Y	Y	6/8
Fernández-Fernández et al. (2015)	Y	Y	Y	Y	N	N	Y	Y	6/8
Ferreira et al. (2020)	Y	Y	Y	Y	N	N	Y	Y	6/8
Haynes and DeBeliso (2019)	Y	Y	Y	Y	N	N	Y	Y	6/8
Maia et al. (2019)	Y	Y	Y	Y	Y	N	Y	Y	7/8
Martínez-Gómez et al. (2019)	Y	Y	Y	Y	N	N	Y	Y	6/8
Maté-Muñoz et al. (2018)	Y	Y	Y	N	N	N	Y	Y	6/8
Maté-Muñoz et al. (2017)	Y	Y	Y	N	N	N	Y	Y	6/8
Sauvé et al. (2024)	Y	Y	Y	Y	N	N	Y	Y	6/8
Tibana et al. (2018)	Y	Y	Y	Y	N	N	Y	Y	6/8
Zeitz et al. (2020)	Y	Y	Y	Y	N	N	Y	Y	6/8

Q1: Were the criteria for inclusion in the sample clearly defined?; Q2: Were the study subjects and the setting described in detail?; Q3: Was the exposure measured in a valid and reliable way?; Q4: Were objective, standard criteria used for measurement of the condition?; Q5: Were confounding factors identified?; Q6: Were strategies to deal with confounding factors stated?; Q7: Were the outcomes measured in a valid and reliable way?; Q8: Was appropriate statistical analysis used?; Y: yes (1 point); N: no (0 points)

**Table 3:** Study quality analysis using the PEDro scale.

Study criteria	Brandt et al. (2022)	Cosgrove et al. (2019)	Yüksel et al. (2018)	Schlie et al. (2023)
Eligibility criteria	Y	Y	Y	Y
Random allocation	Y	Y	Y	Y
Concealed allocation	Y	Y	Y	Y
Baseline comparability	Y	Y	Y	Y
Blinded participants	N	N	N	N
Blinded therapists	N	N	N	N
Blinded assessors	Y	Y	Y	Y
Adequate follow-up	Y	Y	Y	Y
Intention-to-treat analysis	Y	Y	Y	Y
Between-group comparisons	Y	Y	Y	Y
Point estimates and variability	Y	Y	Y	Y
Total score	9/11	9/11	9/11	9/11

Y: yes (1 point); N: no (0 points)

### Biomechanical Variables

The literature on biomechanical variables of CrossFit encompasses a wide range of studies focusing on the measurement and analysis of movement patterns, joint forces, and muscle activity during CrossFit exercises. Table 4 summarizes key biomechanical variables and their measurement tools.

**Table 4a:** Biomechanical variables of evaluation of CrossFit performance.

Authors	Design	Topic	Sample	Analysis	Main Findings
Brandt et al. (2022)	Randomized controlled trial	CrossFit program effects on physically inactive employees	Sedentary behavior participants (39 in the intervention group and 31 in the control group)	Changes in mobility, strength (maximum isometric strength in kg), well-being (WHO-5 score), and back issues (pain intensity, limitation, and frequency) were assessed before and after 6 months of CrossFit training.	Significant effects were found for mobility and strength, but not for well-being.
Brognara et al. (2023)	Cross-sectional	Stability and postural balance	42 CrossFit athletes	Custom foot orthoses were used for postural/balance assessment. Static and dynamic tests (overhead squats and pistol squats) were performed before and after using different insoles. IMU for postural balance evaluation was used.	In the pistol squat, both orthotic therapies were significantly lower than the average of the oscillations from the first use, with a stable improvement. The overhead squats and pistol squat tests proved a statistically significant benefit.
Butcher et al. (2015b)	Cross-sectional	Relationship between physiological measurements and CrossFit performance	14 CrossFit athletes	Anaerobic power (minimum, average, and peak) in different WODs.	No relationships were found between benchmark WODs (Grace and Fran) and laboratory test variables. The CrossFit Total was the sole predictor of performance in these workouts.
Cejudo (2022)	Cohort study	Optimal upper-limb range of motion for performance	26 CrossFit athletes	Shoulder, elbow, and wrist passive ROM were measured via an inclinometer. Clean technique at 80% 1RM was evaluated initially and after 7 months.	The study found that shoulder external rotation (≥ 123°), elbow pronation (≥ 112°), and wrist extension (≥ 90°) were key for proper clean technique, with a > 85% success rate. Limited ROM increased technical errors and injury risk, highlighting the need for targeted flexibility training in CrossFit.
Cosgrove et al. (2019)	Randomized controlled trial	High-intensity functional training effects	45 participants (men and women) with 0–27 months of high-intensity functional training experience	Participants completed a six-month CrossFit program. Flexibility, power, muscular endurance, and strength were tested before and after the program across three sessions.	All fitness domains improved in women over time, with greater cardiorespiratory gains in the 0–6-month group. In men, flexibility, muscular endurance, and strength showed significant improvements.
Ferreira et al. (2020)	Cross-sectional	Movement efficiency and technical execution	10 trained CrossFit athletes	Joint angles, bar path, concentric phase velocity	Fatigue during deep squats induced significant interquartile variability in biomechanics, with changes in joint coordination and barbell velocity, emphasizing technique degradation under fatigue.

1RM: one-repetition maximum; IMU: Inertial measurement unit; WHO-5 Index: World Health Organization Well-Being Index; WOD: workout of the day

**Table 4b:** Biomechanical variables of evaluation of CrossFit performance.

Authors	Design	Topic	Sample	Analysis	Main Findings
Haynes and DeBeliso (2019)	Cross-sectional	Relationship between CrossFit performance and handgrip strength	15 female participants of varying experience levels	CrossFit performance was assessed with a WOD of 3 rounds, each consisting of 30 s at four stations: fan bike (max calories), air squats, sit-ups, and burpees (max reps), followed by 2 min and 30 s of rest.	Sit-up performance showed a moderately positive significant relationship with handgrip strength (r = 0.44).
Maté-Muñoz et al. (2018)	Cross-sectional	Muscular fatigue responses	32 strength-trained males	Jump height, average power, and maximum take-off velocity during a CMJ (pre- and post-WODs)	The “Cindy” and power clean WODs induced significant losses in jump height (7.3%), take-off velocity (13.8%), relative (4.6%), and average power (4.2%).
Martínez-Gómez et al. (2019)	Cross-sectional	Relationship between strength and power during the full-squat exercise and CrossFit performance	20 trained males	The full squat test used an incremental free-weight protocol, measuring bar velocity with a linear transducer. 1RM was estimated via load-velocity interpolation. Five WODs assessed performance, evaluating 1RM, peak, and mean power.	Squat variables showed moderate to strong correlations (r = 0.47–0.69) with WOD performance. Absolute and relative 1RM and relative power were key predictors. Significant group differences were found in strength and power metrics, highlighting their value in assessing CrossFit performance.
Sauvé et al. (2024)	Cross-sectional	Physical profile of elite CrossFit athletes	16 elite CrossFit athletes	1RM strength (squat, bench press, deadlift, clean and jerk), CMJ, SJ, peak power	Elite athletes demonstrated high 1RM values and power output, with CMJ height ~44.7 cm and relative back squat 1RM ~2.0× body mass.
Yüksel et al. (2018)	Randomized controlled trial	Effects of CrossFit on strength	32 trained wrestlers	Cindy was performed 3 times a week for 8 weeks, consisting of AMRAP of 5 pull-ups, 10 push-ups, and 15 air squats in 20 min. An accelerometric system measured squat jump height and bench press force, with the latter assessed using repetitive tests at 30% 1RM.	Cindy’s WOD induced greater improvements than classic wrestling training on squat performance, vertical jump height, bench press force, and bench press bar velocity.
Zeitz et al. (2020)	Cross-sectional	Determinants of CrossFit performance	22 recreationally trained individuals	1RM back squat, deadlift, overhead press, and performance on benchmark “Fran” and workout 19.1.	All strength variables showed moderate to strong positive correlations with WOD performance, indicating higher CrossFit performance with greater strength. Fatigue tolerance might influence 19.1 performance due to maximal effort demands. The CrossFit Total workout (maximum strength) was key for optimal performance in time-to-completion tasks with absolute loads.

1RM: one-repetition maximum; AMRAP: as many rounds as possible; CMJ: countermovement jump; WOD: workout of the day

A growing number of studies have explored the biomechanics of key CrossFit exercises, including Olympic lifts like the clean and jerk and snatch, as well as various gymnastic movements (Cejudo, 2022; Martínez-Gómez et al., 2019). These investigations commonly relied on specialized tools to assess performance and movement quality. For example, motion capture systems have been used to analyze joint angles and movement patterns in dynamic tasks (Cejudo, 2022). On the other hand, force platforms have provided insights into ground reaction forces and power output during loaded lifts and jumps (Martínez-Gómez et al., 2019; Maté-Muñoz et al., 2018; Yüksel et al., 2018). Additionally, Ferreira et al. (2020) applied surface EMG and kinematic analysis to examine neuromuscular fatigue and interquartile variability in movement patterns during deep squats in CrossFit athletes, showing that fatigue significantly altered the bar path and joint coordination. Drawing from these methodologies, we grouped the studies according to their primary objectives: i) experimental interventions incorporating CrossFit training; ii) correlation-based analysis linking biomechanical factors to performance; iii) investigations into the acute responses to different CrossFit training formats; and iv) other biomechanical approaches aimed at understanding movement efficiency and injury risk.

#### CrossFit as a Training Intervention

Brandt et al. (2022) analyzed the effect of six months of CrossFit training (2 sessions of 60 min per week) in inactive adults. The results showed consistently large positive effects on mobility and strength. The authors concluded that health professionals should consider CrossFit a safe, efficient, and applicable training concept for individuals at risk of developing chronic diseases due to inactivity and sedentary behavior. Cosgrove et al. (2019) examined the effectiveness of a CrossFit program after six months in participants (men and women) with different high-intensity functional training experiences. That study showed a positive effect of CrossFit program participation on multiple fitness domains, with greater improvements for women with less experience. Yüksel et al. (2018) investigated the effects of CrossFit training on jump and strength variables in healthy men. The experimental group underwent CrossFit training three times a week for eight weeks using Cindy WOD. The findings were consistent with previous research showing the benefits of CrossFit training to physical fitness performance. Studies consistently highlighted the positive effects of CrossFit interventions on fitness performance, mobility, and strength across diverse populations.

#### Correlational Studies in CrossFit Performance

Haynes and DeBeliso (2019) studied the relationship between CrossFit performance and handgrip strength in women because this variable had been identified as a good predictor of total body strength and functional ability in CrossFit and non-CrossFit practitioners (DeBeliso et al., 2015). The results only showed a moderately significant relationship between handgrip strength and sit-up performance. The study conducted by Martínez-Gómez et al. (2019) explored the relationship between CrossFit performance and power and strength variables measured in the full-squat exercise. Performance in different WODs was also measured, and overall CrossFit performance was determined. Results showed positive correlations between squat variables and performance in the WODs, with overall CrossFit performance strongly associated with the 1RM and mean and peak power obtained in the full-squat exercise. That study suggested that strength and power variables measured in the full-squat positively related to CrossFit performance, indicating that the squat exercise could predict lower-limb muscle movements in CrossFit. The study by Martínez-Gómez et al. (2019) highlighted the importance of analyzing strength variables in assessing athletic performance, with potential implications for predicting success in CrossFit. Similarly, Sauvé et al. (2024) provided normative data on elite CrossFit male athletes, reporting 1RM back squat values equivalent to ~2 times body mass, CMJ heights of nearly 44 cm, and peak power output exceeding 800 W. Additionally, Zeitz et al. (2020) evaluated the 1RM back squat, the deadlift, and the overhead press, and the sum of the three exercises was determined as the CrossFit Total. Results showed that all strength variables were correlated (moderate to strong) with performance in CrossFit workouts like “19.1” and “modified Fran”, indicating greater CrossFit performance with greater 1RM values. In the same line, Butcher et al. (2015b) analyzed different WODs on separate days and CrossFit Total (1RM back squat, overhead press, and deadlift). Only whole-body strength could partially explain the performance in Grace and Fran WODs, although the anaerobic threshold was also associated with sports performance. Correlational studies highlighted the predictive value of specific strength and power metrics for CrossFit performance.

#### Acute Effects of CrossFit Training

Maté-Muñoz (2018) assessed the cardiometabolic and muscular fatigue responses to different CrossFit workouts. The workouts resulted in elevated heart rates, the RPE, and blood lactate levels, indicating vigorous exercise intensity. Muscular power losses were observed after the workouts. That study highlighted the importance of quantifying exercise intensity for proper training load prescription to reduce the risk of injury and optimize performance.

#### Movement Efficiency and Technical Execution

Cejudo (2022) analyzed the influence of the range of motion of the upper limb’s joints on the technical execution and performance of the power clean exercise. That author observed that athletes with a greater range of motion in shoulder external rotation, elbow pronation, and wrist extension were more likely to execute the power clean movement correctly. Building on this, Martínez-Gómez et al. (2019) highlighted the importance of lower-body power by showing a strong association between squat-derived power output and CrossFit performance (r = 0.47–0.69) among 20 trained males. Athletes who produced higher peak and mean power output in the full squat tended to perform better in WODs, reinforcing that efficient force production contributed significantly to overall performance. In addition to strength and mobility, control and balance also appeared to be crucial. Brognara et al. (2023) assessed postural stability in CrossFit athletes and found that better balance, especially during demanding tasks such as the overhead squat and the pistol squat, was linked to improved movement control. These insights suggested that minimizing compensatory movements through better postural alignment could enhance technical execution and help prevent injury.

#### Injury Incidence and Risk Factors

Several studies have investigated injury risk and mechanisms in CrossFit training (Gardiner et al., 2020; Rodríguez et al., 2022), aiming to identify vulnerabilities in specific movements and inform safer training practices. The shoulder (26%), the spine (24%), and the knee (18%) emerged as the most affected regions, likely due to the mechanical demands of overhead lifts, spinal loading, and high-impact plyometrics. The injury incidence in individual studies ranged widely from 12.8% to 73.5%, while reported rates varied between 0.27 and 3.3 per 1,000 training hours. Beyond incidence figures, biomechanical studies offered insights into modifiable risk factors. Cejudo (2022) found that athletes with limited shoulder, elbow, or wrist mobility were more likely to perform the power clean with technical errors, increasing their risk of injury. Similarly, Brognara et al. (2023) demonstrated that postural stability during complex movements like the overhead squat and pistol squat might protect against injury by reducing compensatory patterns. Additionally, Brandt et al. (2022) observed that CrossFit training reduced back pain in previously sedentary individuals, suggesting that, when properly implemented, CrossFit might also serve as a corrective modality for musculoskeletal discomfort.

These results suggest that CrossFit has a relatively low injury risk and highlight factors like training frequency, duration of CrossFit experience, and the level of competition as important factors in the injury incidence and incidence rates. Finally, researchers have investigated potential performance-enhancing techniques, such as kinematic and kinetic strategies, to optimize training protocols and improve athletic performance in CrossFit. For example, when developing exercise-specific force, the exercise should be completed closer to set failure with fewer repetitions, which can be achieved using complex or high-volume contrast training to pre-fatigue the lighter exercise. When the objective is to improve velocity for the target exercise, it can be combined with a heavier contrast pair to create a post-activation performance-enhancing effect. Alternatively, cluster set designs can be used to maintain high velocities and reduce drop-off, and traditional training is the most effective for increasing the 1RM squat (Marshall et al., 2021; Sauvé et al., 2024).

### Physiological Variables

Table 5 shows the most commonly included variables to determine CrossFit’s performance, including VO_2max_, blood lactate concentration, HRV, the RPE, blood and salivary samples, and body composition.

**Table 5a:** Physiological variables of evaluation of CrossFit performance.

Authors	Design	Topic	Sample	Analysis	Main Findings
Butcher et al. (2015a)	Cross-over	Relative intensity of two types of CrossFit exercise	10 trained CrossFit participants	Heart rate, rating of perceived exertion, session duration, and relative intensity	Both workout formats (circuit and HIIT) elicited high cardiovascular and perceptual responses; HIIT WODs produced higher relative intensity compared to circuit-style WODs.
Butcher et al. (2015b)	Cross-sectional	Relationship between physiological measurements and CrossFit performance	14 CrossFit athletes	VO_2max_, anaerobic threshold, respiratory compensation threshold, and fatigue index were analyzed during different WODs	Laboratory physiological variables showed no relation to benchmark WODs (Grace and Fran). The CrossFit Total positively correlated with performance in both. Whole-body strength and CrossFit Total explained 77% of the variance in Grace and 42% in Fran, but did not impact Cindy’s performance.
Faelli et al. (2020)	Randomized controlled trial	Physiological responses to CrossFit and resistance training	20 moderately trained individuals	Salivary cortisol, interleukin-1β, and uric acid were assessed via ELISA pre- and post-8-week resistance training to compare with CrossFit training	CrossFit produced a more profound catabolic impact than resistance training.
Fernández-Fernández et al. (2015)	Cross-sectional	Physiological responses to CrossFit workouts.	10 trained subjects	VO_2_, respiratory exchange ratio, heart rate, blood lactate concentration, and the RPE were measured before and after each WOD	Moderate differences were found in VO_2_ and %VO_2max_, with higher values in Cindy. Fran had more time above respiratory exchange ratio 1, while Cindy remained below 1. Participants spent 42.2% (Cindy) and 29.3% (Fran) of the workout time above 91% of the maximum heart rate, indicating that training CrossFit 2–3 times/week meets ACSM intensity guidelines for aerobic fitness and fat loss.
Maia et al. (2019)	Case study	Neuromuscular and autonomic responses to a CrossFit competition	3 CrossFit athletes (A, B, and C)	HRV, RPE, CMJ, and internal competition load were evaluated pre- and post-competition	Athlete A showed impaired neuromuscular and autonomic recovery during competition. Athlete C’s CMJ declined throughout the first day. Male athletes had reduced HRV, indicating autonomic suppression. Neuromuscular function decreased by the competition’s end.

ACSM: American College of Sports Medicine; CMJ: countermovement jump; HIIT: high-intensity interval training; HRV: heart rate variability; VO_2max_: maximal oxygen uptake_;_ RPE: rate of perceived exertion; WOD: workout of the day

**Table 5b:** Physiological parameters of evaluation of CrossFit performance.

Authors	Design	Topic	Sample	Analysis	Main Findings
Maté-Muñoz et al. (2017)	Cross-sectional	Muscular fatigue responses to three CrossFit modalities: gymnastics, metabolic conditioning, and weightlifting.	34 healthy subjects	CMJ and blood lactate analyzed before and after each CrossFit modality	CMJ performance declined across all CrossFit modalities. Fatigue recovery followed metabolic conditioning, likely due to rest intervals restoring phosphocreatine. High intensity and volume in gymnastics and weightlifting WODs may reduce muscle-tendon stiffness, prolong the CMJ isometric phase, and impair jump ability.
Maté-Muñoz et al. (2018)	Cross-sectional	Cardiometabolic and muscular fatigue responses against different WODs	32 strength-trained males	Heart rate, RPE, and blood lactate were measured before and after each WOD	RPE and the heart rate were significantly higher for Cindy (WOD 1) than the double under (WOD 2) and power clean (WOD 3) workouts. Blood lactate concentration always exceeded 10 mmol/L, indicating a high intensity of exercise.
Sauvé et al. (2024)	Cross-sectional	Physiological profile of elite CrossFit athletes	16 elite CrossFit athletes	VO_2max_, ventilatory threshold, Wingate test, body composition (DXA)	VO_2max_ ranged from 56 to 64 mL•kg^-1^•min^-1^; Wingate peak power exceeded 800 W in males; body fat content was 11.8% in males and 17.2% in females; aerobic and anaerobic capacities were well-developed in elite CrossFitters.
Schlie et al. (2023)	Randomized controlled trial	CrossFit intervention for enhancement of cardiorespiratory fitness and well-being	16 healthy CrossFit beginner athletes	VO_2max_, WHO-5 Index, body composition, and heart rate reserve pre- and post-intervention	VO_2max_ showed a large improvement, with strong negative correlations between baseline VO_2max_ and its progression after nine months (r = -0.65). Well-being increased by 8.7%, heart rate reserve improved at 1- and 5 minutes post-exercise, and resting metabolic rate rose by 2.2%.
Tibana et al. (2018)	Cross-sectional	Physiological responses to shorter and longer CrossFit training sessions.	9 trained males	Blood lactate, heart rate, and RPE were analyzed during and after a shorter and longer duration CrossFit	Blood lactate concentration was higher after the shorter recovery session (15.9 ± 2.2 mmol/L) compared to the longer session (12.6 ± 2.6 mmol/L; p = 0.019). Blood lactate remained significantly elevated during recovery for both sessions compared to pre-exercise. The RPE increased immediately post-exercise for both sessions, with no significant differences between WODs.

CMJ: countermovement jump; VO_2max_: maximal oxygen uptake_;_ RPE: rate of perceived exertion; WHO-5 Index: World Health Organization Well-Being Index; WOD: workout of the day.

**Table 5c:** Physiological parameters of evaluation of CrossFit performance.

Authors	Design	Topic	Sample	Analysis	Main Findings
Zeitz et al. (2020)	Cross-sectional	Physiological determinants of CrossFit performance	22 recreationally trained individuals	VO_2max_, ventilatory thresholds, and body composition were analyzed, as well as the performance on the benchmark “Fran” and workout 19.1.	VO_2max_ significantly predicted performance in 19.1 workouts. All strength variables showed moderate to strong positive correlations with CrossFit performance. Fatigue tolerance might also play a key role in 19.1 performance.

VO_2max_: maximal oxygen uptake

#### Maximal Oxygen Uptake

Butcher et al. (2015b) analyzed the influence of physiological variables on predicting CrossFit performance in specific WODs. Those authors concluded that CrossFit benchmark WOD performance did not correlate significantly with VO_2max_, Wingate power/capacity, respiratory compensation, or anaerobic thresholds. Therefore, in addition to their typical training, CrossFit athletes should likely ensure adequate strength and aerobic endurance to optimize performance on at least some benchmark WODs. Schlie et al. (2023) investigated the long-term effects of a CrossFit program in 16 healthy beginner participants. The training program lasted nine months, with two sessions per week. After CrossFit training, significant improvements in VO_2max_ (11.5%) and overall well-being (8.7%) were observed, with a significantly large negative correlation between baseline VO_2max_ and performance improvement (r = −0.65), indicating greater adaptations in less conditioned individuals. Therefore, while VO_2max_ continues to serve as a key indicator of aerobic capacity, its role in CrossFit is nuanced, requiring a blend of strength and endurance for optimal results.

#### Acute Responses to WODs

Butcher et al. (2015a) compared the acute responses to two CrossFit training formats: circuit-based and high-intensity interval workouts. Both elicited vigorous cardiovascular and perceptual responses (e.g., near-maximal HR and RPE values), but the HIIT-style session produced significantly higher relative intensity.

Fernández-Fernández et al. (2015) examined the acute physiological effect of two WODs (“Fran” and “Cindy”) in male and female individuals of different performance levels. Both WODs could be characterized as high-intensity workouts, achieving near maximal physiological (e.g., 90–95% of the maximum heart rate) and perceptual responses (e.g., RPE values > 8) in all participants. Tibana et al. (2018) compared the heart rate, blood lactate concentration and the RPE in short- (~4 min) and long-duration (~17 min) CrossFit sessions. Results showed similarities in the maximum heart rate between both protocols, whereas blood lactate concentration was greater in short- compared to long-duration CrossFit sessions. This information could be relevant to determine the type of physiological stress induced in an athlete according to duration and intensity applied during the session, to organize the efforts during a training program. Acute physiological responses highlight the high-intensity nature of CrossFit and the need for careful session structuring to manage stress and fatigue.

Studies conducted by Maté-Muñoz et al. (2017, 2018) analyzed the cardiometabolic and muscular responses to different CrossFit workouts. Those authors found high correlations between blood lactate concentration and the average heart rate in different WODs (r = 0.92–0.94). Maia et al. (2019) analyzed internal and external load variables in male CrossFit practitioners during competition. Participants performed five events: bodyweight exercises, Olympic weightlifting, and aerobic activities. The results indicated that two CrossFit competitions on consecutive days negatively influenced the neuromuscular and autonomic function of the male practitioners. Based on these results, trainers and exercise professionals must be cautious when prescribing high-intensity training. Any training with high-intensity exercise, including recovery periods, could be essential to avoid muscle fatigue and decrease the likelihood of injury. In addition, coaches must be sure that individuals assigned to any CrossFit program are free of any cardiovascular or respiratory conditions or injuries that could jeopardize their health.

#### Competitive Stress and Long-Term Adaptations

Faelli et al. (2020) compared the catabolic and cardiorespiratory responses of CrossFit and resistance training in moderately trained males. Twenty participants were randomly assigned to a CrossFit group (n = 10; 30 min/day of WOD) or a resistance training group (n = 10; 30 min/day of resistance exercises) for three weeks. Salivary biomarkers, such as cortisol, interleukin-1β, and uric acid, were significantly elevated in the CrossFit group post-intervention, suggesting a higher acute catabolic effect. Additionally, VO_2max_ and ventilatory thresholds improved significantly in both groups, though the CrossFit group exhibited a slightly greater effect size. No significant changes in body composition were reported for either group across the short intervention period. CrossFit induced greater catabolic responses than resistance training. After prolonged CrossFit engagement, Schlie et al. (2023) highlighted long-term physiological benefits, including VO_2max_ enhancements and improved systemic efficiency. CrossFit’s adaptive potential is evident in competitive and recreational contexts, with long-term physical and mental health benefits.

## Discussion

This systematic review analyzed the biomechanical and physiological variables related to physical performance in CrossFit. The most frequently evaluated biomechanical variables included movement velocity, force and power output, movement technique, vertical jump performance (e.g., CMJ), and maximum dynamic strength (e.g., 1RM). Considering the physiological aspect, the literature most often assessed VO_2max_, blood lactate concentration, HRV, and the RPE. These measures reflect the dual demands of CrossFit workouts, which blend resistance and metabolic conditioning elements.

Despite the increasing popularity of CrossFit, the number of studies assessing its biomechanical and physiological demands remains limited, particularly among female participants. For example, Schlegel and Křehký (2022) examined sex-based differences in performance at the CrossFit Games, while Mangine et al. (2023) reported that repeated participation in CrossFit-Open workouts led to notable performance improvements, especially among women. Similarly, Kićanović et al. (2022) found that CrossFit training produced greater improvements in body composition than traditional gym-based training in active males, thereby reinforcing CrossFit’s utility across diverse populations.

Compared to other modalities, CrossFit appears to elicit physiological adaptations like those seen in high-intensity interval training (HIIT) and traditional resistance programs. Milanović et al. (2015) showed that HIIT significantly improved VO_2max_, while Schoenfeld et al. (2017) demonstrated the efficacy of heavy-load resistance training in increasing strength and hypertrophy. In CrossFit, the combination or integration of these components offers a hybrid model capable of improving cardiovascular fitness, anaerobic capacity, and body composition across training levels (Gianzina and Kassotaki, 2019; Mangine et al., 2020). Furthermore, due to its time-efficient design, characterized by short rest periods, high power output, and multi-joint movements, CrossFit may offer practical advantages over more time-consuming traditional exercise formats (Claudino et al., 2018; Fernández-Fernández et al., 2015). Notably, HIIT has also been shown to improve cognitive domains such as inhibitory control and working memory (Yue et al., 2025). Given its structural similarities to HIIT, CrossFit might provide similar cognitive benefits, though this hypothesis needs to be directly tested.

Beyond identifying the most studied performance variables, it is critical to interpret what these findings imply for training practices. Movement velocity and power output, for instance, which are strongly correlated with neuromuscular fatigue, were widely assessed in the reviewed studies (González-Badillo et al., 2022; Sánchez-Medina and González-Badillo, 2011). Prior research has shown that reductions in movement velocity, particularly during resistance training, can serve as reliable proxies for internal fatigue and metabolic stress (Pareja-Blanco et al., 2020; Sánchez-Medina and González-Badillo, 2011). Although this approach has not yet been applied directly in CrossFit, the concept holds promise as a practical, non-invasive method to monitor training intensity and recovery during WODs. From a programming standpoint, velocity-based resistance training could help coaches fine-tune training loads and reduce injury risk due to overreaching, especially during high-volume or high-intensity phases. To advance this line of inquiry, future longitudinal studies should examine the magnitude of velocity loss during different WOD formats, such as “Cindy” and “Fran”, to analyze the degree of neuromuscular fatigue as well as the recovery pattern over time. Additionally, future studies should seek to validate velocity-based fatigue monitoring by comparing it with EMG markers of neuromuscular function during and after CrossFit workouts.

Vertical jump performance, particularly through CMJ testing, also emerged as a standard metric of lower-body power and fatigue. Among the included studies, CMJ performance consistently declined following intense WODs, validating its use as an acute fatigue marker. Its reliability and non-invasive nature makes the CMJ a suitable tool for assessing readiness or adjusting training prescriptions in real time. For instance, coaches might delay maximal efforts or reduce volume on days when CMJ performance is compromised.

Similarly, 1RM strength testing was prevalent, particularly in Olympic lifts and squats. Findings indicate that CrossFit can yield meaningful strength adaptations, especially among experienced athletes (Bellar et al., 2015). These results are consistent with outcomes seen in traditional resistance training literature (Schoenfeld et al., 2017), affirming the value of incorporating structured strength components within WOD programming. These findings point to the importance of integrating joint mobility, strength, and stability assessments into CrossFit training. Doing so may improve both performance quality and injury resilience during complex, high-load exercises.

From a physiological standpoint, VO_2max_ improvements were well documented across the reviewed studies and consistent with gains seen in HIIT protocols (Milanović et al., 2015). These results support the aerobic efficacy of CrossFit, particularly in novice and recreationally trained populations. Additionally, blood lactate levels and the RPE were consistently elevated post-WOD, indicating high anaerobic and perceptual demands. These responses underline the need for careful recovery planning and suggest that fatigue management should be a key consideration in WOD sequencing. Meanwhile, HRV data, though limited, pointed to autonomic disturbances following high-intensity efforts, reinforcing the importance of tracking recovery in athletes exposed to frequent WODs. These findings align with observations from Seo et al. (2024), who reported sex-based differences in HRV and vascular function following HIIT, suggesting that autonomic adaptations may be influenced by sex.

Findings from this review also highlight differences in training adaptations between novice and experienced CrossFit athletes. Individuals with more training experience consistently demonstrated superior aerobic and anaerobic performance (Bellar et al., 2015; Butcher et al., 2015a), and greater familiarity with CrossFit-specific tasks appeared to enhance the training effect (Zeitz et al., 2020). On the other hand, beginners may experience less pronounced improvements initially, potentially due to the high fatigue levels induced by typical WODs. For this population, it may be advisable to implement lower-intensity sessions early on and progressively increase volume and intensity. Coaches are encouraged to support and educate novices through this adaptation phase to ensure long-term adherence and benefits (Schlegel and Křehký, 2022). Future investigations should also consider sex-based differences in physiological and biomechanical responses to CrossFit training. Recent evidence has demonstrated sex-specific physiological adaptations to CrossFit training. For instance, Barreto et al. (2024) reported differential hematological reactions between men and women following 24 weeks of CrossFit, reinforcing the importance of considering sex-based factors when analyzing training adaptations. Stratified analyses by sex may clarify how males and females differ in adaptation patterns, performance output, and injury susceptibility, enabling more precise training recommendations.

It is important to acknowledge the methodological variation across included studies. Experimental designs generally scored highly on the PEDro scale, supporting the reliability of their findings. In contrast, cross-sectional studies varied slightly in methodological quality based on the JBI checklist, particularly in aspects like participants’ selection and confounder control. While no studies were excluded due to low quality, these differences might affect the consistency of observed effects and should be considered when interpreting the results. The current review also included diverse populations (trained vs. untrained; males vs. females), study designs (cross-sectional vs. longitudinal), and outcome variables. While this heterogeneity reflects the breadth of CrossFit research, it also limits the direct comparability of findings. For example, strength and endurance outcomes may differ substantially between trained and novice athletes, and physiological responses can vary by sex. A more stratified analysis of CrossFit’s effects, by training level and sex, is needed to provide tailored recommendations and deepen our understanding of its efficacy.

Altogether, these insights highlight the multifactorial nature of performance in CrossFit, where physiological readiness, technical proficiency, and recovery management converge. Future research that incorporates sex-specific analysis, real-time monitoring, and standardized fatigue assessments will be essential for refining CrossFit training strategies. Moreover, the growing availability of wearable technologies presents a promising avenue for monitoring movement quality and fatigue in real time. Future research should investigate the validity of using accelerometers, gyroscopes, and force-sensing devices to detect technique breakdowns, track loading patterns, and optimize feedback during CrossFit workouts.

### Limitations

The literature revealed that CrossFit training is important in athletes’ physical performance. The present review revealed important information about the variables that may better explain CrossFit training and competition performance. However, it is important to highlight that results of this review present several limitations. First, no meta-analysis was conducted. Due to the considerable heterogeneity in the study design, outcome measures, and participants’ characteristics, a meta-analysis was not feasible. Although a narrative synthesis was used to integrate findings, the absence of a formal effect direction summary limits the ability to quantify overall effect trends. Future reviews might explore subgroup analysis or stratified meta-analysis, where data allow for such an approach. Second, the methodological quality of the included studies was assessed using the PEDro scale (for randomized controlled trials) and the JBI checklist (for cross-sectional studies). Although no studies were excluded due to low quality, issues such as small sample sizes, limited use of control groups, and lack of blinding may have affected the internal validity and consistency of results. Third, the studies included in this review encompassed a broad range of participants, including novice and experienced athletes, males and females, and recreational versus competitive populations. This population heterogeneity limits generalizability and makes it difficult to draw specific conclusions applicable to all subgroups. Performance improvements and physiological adaptations were often more pronounced in experienced athletes, suggesting that training status should be more clearly accounted for in future analysis. Finally, female participants were underrepresented in several of the reviewed studies, reflecting a broader imbalance in CrossFit research. Given the emerging evidence on sex-based differences in training responses and injury patterns (Mangine et al., 2023; Schlegel and Křehký, 2022), this underrepresentation limits the applicability of findings to female athletes. This underrepresentation of female athletes is particularly limiting given emerging evidence of sex-specific adaptations. Barreto et al. (2024), for instance, reported differential hematological responses between men and women after prolonged CrossFit training, underscoring the necessity of sex-stratified research to develop tailored training recommendations. Increasing female inclusion is essential to ensure more inclusive and generalizable insights. Despite these limitations, this review provides a detailed synthesis of current knowledge and identifies key biomechanical and physiological markers relevant to CrossFit performance, monitoring, and programming.

### Practical Implications

Understanding the biomechanical and physiological demands of CrossFit is essential for several practical applications across coaching, performance optimization, and injury prevention. Based on the findings of this review, the following implications should be emphasized:

i) Optimizing athletic performance. Performance-related variables such as the CMJ, the 1RM load, and force or power output were among the most frequently measured in the literature. These key performance metrics can be used to inform individualized resistance training programs. For example, monitoring CMJ performance across resistance training cycles may provide insight into neuromuscular readiness and fatigue status, while monitoring 1RM trends helps assess strength development and program effectiveness.
ii) Injury prevention and rehabilitation enhancement. Several studies identified the shoulder, the spine, and the knee as the most frequently injured areas in CrossFit athletes (Gardiner et al., 2020; Rodríguez et al., 2022). Cejudo (2022) further demonstrated that a limited range of motion in the upper limbs, particularly in shoulder external rotation and wrist extension, negatively impacted technical execution in movements such as the power clean. Identifying these biomechanical deficits early can aid in correcting movement patterns, reducing injury risk, and supporting safer rehabilitation processes.
iii) Improved training load management. Physiological markers such as blood lactate concentration and HRV were shown to fluctuate significantly after WODs (Maté-Muñoz et al., 2018; Tibana et al., 2018). These variables provide valuable real-time data on the internal load and recovery needs. Coaches can use these insights to regulate session intensity better, implement recovery strategies, and avoid overtraining, especially in high-volume or competition phases.
iv) Enhancing coaching strategies and feedback. A deeper understanding of movement velocity, power development, and fatigue progression allows coaches to provide more effective and individualized feedback during training. For instance, using velocity loss as a proxy for neuromuscular fatigue may help coaches adjust work-rest ratios or exercise prescriptions on the spot.
v) Integration of evaluation and monitoring tools. The use of tools such as the CMJ, movement velocity recording, lactate concentration measurements, and technical movement assessments can be integrated directly into the training environment to monitor progress and adjust programming dynamically. These tools can help coaches and practitioners make evidence-informed decisions and create more responsive training cycles aligned with the athlete’s current state.

## Conclusions

This systematic review examined the biomechanical and physiological variables most frequently assessed in CrossFit training and competition. Based on the included studies, the following conclusions can be drawn:

- CrossFit training, like other forms of high-intensity training, is associated with improvements in VO_2max_, muscle strength, hypertrophy, and muscle endurance, while contributing positively to body composition, particularly through reductions in fat mass and increases in lean body mass (Kićanović et al., 2022; Mangine et al., 2023) (Table 5).
- When training volume, relative load, and technical execution are adequately managed, CrossFit can be a safe and effective method of improving fitness in healthy adults, including recreational practitioners and beginners seeking a diverse and challenging workout routine.
- Biomechanical and physiological variables are commonly used to assess performance and fatigue. The most frequently measured biomechanical markers included movement velocity, force and power output, the CMJ, movement technique, and 1RM strength, as detailed in Table 4. On the physiological side, blood lactate concentration, VO_2max_, HRV, and RPE were the most reported (Table 5).
- Several studies indicated strong correlations between strength/power indicators (e.g., 1RM, CMJ, velocity loss) and CrossFit performance, particularly in benchmark workouts. In addition, movement technique and postural control, such as range of motion in complex lifts like the power clean, also played a role in performance outcomes, as demonstrated by Cejudo (2022).
- The combined aerobic and anaerobic demands of CrossFit training promote cardiovascular adaptations while simultaneously inducing substantial physiological stress. This aspect was reflected in elevated blood lactate concentration during WODs and in shifts in HRV and RPE scores post-exercise (Maté-Muñoz et al., 2018; Tibana et al., 2018), highlighting the importance of these variables for fatigue monitoring and recovery planning.
- Experienced CrossFit practitioners tend to display greater aerobic capacity, anaerobic power, and training adaptability compared to novices, likely due to greater familiarity with movement standards and competition demands (Mangine et al., 2023; Zeitz et al., 2020). This evidence reinforces the importance of progressive training design for beginners to avoid early fatigue and improve long-term adherence.

Overall, this review reinforces the multidimensional nature of CrossFit, which integrates physiological conditioning with complex movement patterns and variable intensity. These features contribute to improvements in multiple aspects of physical performance, provided the training is structured, individualized, and monitored appropriately.
